# The effect of non-local coupling of fibroblasts on pacing dynamics in a 2D tissue: a simulation study

**DOI:** 10.1038/s41598-025-99674-6

**Published:** 2025-05-08

**Authors:** S. Sridhar, Richard H. Clayton

**Affiliations:** School of Computer Science and Insigneo Institute for in-silico Medicine, University of Sheffield, Sheffield, UK

**Keywords:** Biomedical engineering, Computational models, Biological physics

## Abstract

Although myocytes in healthy hearts are usually coupled to nearest neighbours via gap junctions, under conditions such as fibrosis, in scar tissue, or across ablation lines, myocytes can uncouple from their neighbours. However it has been experimentally observed that electrical conduction can still occur across these uncoupled regions via fibroblasts. In this paper we propose a novel model of non-local coupling between myocytes and fibroblasts in a 2*D* tissue, and hypothesise that such long-range coupling can give rise to pro-arrhythmic re-entrant wave dynamics. We have simulated the scar and the surrounding border zone via simultaneous coupling of fibroblasts with both proximal and distal regions of myocardium. We find that in this setup the border zone itself is a dynamical outcome of the coupling between cells within and outside the scar. We have determined the effect of the border zone on the stability of waves generated by rapid pacing. Furthermore we have identified key parameters that determine wave dynamics in this geometry, and have also described the mechanism underlying the complex wave dynamics. These findings are of significance for our understanding of cardiac arrhythmias associated with regions of myocardial scar.

## Introduction

The heart is a syncytium where coordinated mechanical contraction is enabled by the propagation of synchronised waves of electrical excitation. Myocytes and fibroblasts constitute two of the most important cell types in the mammalian heart. Myocytes, which are typically larger than fibroblasts, are responsible for the functional behaviour of the heart, supporting the initiation and propagation of the electrical activity that results in synchronized contraction. The smaller but more numerous fibroblasts act to maintain the structural integrity of the heart^1^ and do not influence the electrophysiology of the myocytes. In injured or diseased hearts the fibroblasts differentiate into much larger myofibroblasts. These myofibroblasts play a crucial role in the repair of heart muscles^1,2^. In aged or diseased hearts the number of fibroblasts and myofibroblasts may increase substantially (up to 40 percent^3^) resulting in increased collagen deposition causing fibrosis, which in turn affects electrical coupling and propagation of the action potential. Note that although we have used the terms fibroblast and myofibroblast interchangeably in rest of the paper, as we are modelling a fibrotic tissue observed in injured or ageing hearts we have only considered the electrical interactions between myocytes and myofibroblasts.

While the possibility of electrical coupling between myocytes and fibroblasts (*M*–*F* coupling) has been debated for a long time^2,4^, more recent studies have confirmed that fibroblasts can indeed be coupled to myocytes via gap-junctions^5,6^. Experiments have shown that coupling between myocytes and fibroblasts can significantly alter the conduction properties of the tissue^7,8^. Further *M*–*F* coupling is also known to modify the excitability^9^ and resting membrane potential^10^ of myocytes. In both tissue and organ, fibrosis has been observed to affect wave propagation and create a substrate for cardiac arrhythmia^11–16^.

The mechanisms by which fibroblasts can modify electrical activity in healthy and diseased myocytes and tissue have been explored in several in silico studies^17–23^. These studies typically represent either fibroblasts coupled to single myocyctes, or fibroblasts embedded within simulated tissue thereby electrically coupling nearest myocytes. However both cell-culture and in vivo studies have suggested that fibroblast mediated coupling may enable action potential propagation between otherwise uncoupled myocytes^2,7,24^. Heterocellular cell culture experiments have shown that fibroblast inserts can enable electrotonic conduction between myocytes upto 300 $\upmu$m apart^7^. Electron microscope based reconstructions have suggested that in the sino-atrial node an individual fibroblast can form membrane juxtaposition with nearby myocytes covering up to 720  $\upmu \textrm{m}^2$^24^. In vivo, fibroblasts have been observed to form large sheet-like extensions having additional folds and elongated cytoplasmic processes^24–26^ and are estimated to cover a total surface area of 1500 $\upmu \textrm{m}^2$^24,25^. Fibroblasts that have such long extensions can potentially couple with multiple myocytes that are spatially distant. It is plausible to expect that, if present in vivo, such long-range interactions between distant myocytes mediated via fibroblasts have the potential to modify tissue electrophysiology and dynamics by changing conduction and recovery properties of the medium. Injured or diseased hearts have a proliferation of larger myofibroblasts that could then increase the possibility of such long-range coupling. Furthermore, non-local coupling via *M*–*F* links can occur across ablation lines, producing conduction pathways between electrically isolated regions of tissue^27^. Such complex conduction pathways might also occur when islands of myocytes are trapped in a sea of fibroblasts^28,29^.

In our earlier study we developed 2-cell motifs to investigate the effect of non-local gap-junctional coupling on mutually uncoupled myocytes via active fibroblasts in computational models^30^. We identified regimes of myocyte dynamics that depended on gap-junctional conductance strength, the *M*–*F* connection topology, and parameters of the myocyte and fibroblast models.

In the present study we have implemented on a 2*D* domain *M*–*F* links that electrically connect diffusively uncoupled regions of tissue. We hypothesise that such non-local *M*–*F* coupling can modify the electrical properties of the tissue and promote the onset of reentrant waves during pacing. We have described the formation of dynamical border zones around a scar due to the interaction of spatially separate regions via non-local *M*–*F* links. We have identified the *M*–*F* link parameters that can give rise to such dynamical border zones and subsequently create a region of conduction block followed by reentrant wave formation. Furthermore we have described the mechanism that gives rise to conduction block in terms of electrical properties of the tissue.

## Methods

### Cell models

The electrical activity of myocytes was described using the *TNNP-TP06* model of human ventricular cells^31,32^, while the electrophysiological properties of the fibroblasts were described using the *MacCannell* “active” fibroblast model^33^. The time variation of the transmembrane voltage *V* for myocytes coupled to *np* fibroblasts was described as,

1 $$\begin{aligned} C_m \times \frac{dV}{dt} = -I_{ion} + \sum _{i=1}^{np} Gs (V_{fi} - V) \end{aligned}$$

Here $I_{ion}$ is the total of all ionic currents:

2 $$\begin{aligned} I_{ion}= I_{Na} + I_{to} + I_{K1} + I_{Kr}+ I_{Ks} + I_{CaL}+ I_{NaCa} + I_{NaK} + I_{pCa} + I_{pK} + I_{bCa} + I_{bNa} \end{aligned}$$

where $I_{Na}$ is the sodium current, $I_{to}$ is the transient outward current, $I_{K1}$, $I_{Kr}$ and $I_{Ks}$ are the inward rectifier, delayed rectifier and slow delayed rectifier potassium currents, $I_{CaL}$ is the L-type $Ca^{2+}$ current, $I_{NaK}$ is the $Na^{+}/K^{+}$ pump current, $I_{NaCa}$ is the $Na^{+}/Ca^{2+}$ exchanger current, $I_{pCa}$ and $I_{pK}$ plateau calcium and potassium currents and $I_{bCa}$ and $I_{bNa}$ are the background $Na^{+}$ and $Ca^{2+}$ currents. $V_{fi}$ is the transmembrane potential of the *i*th fibroblast while *Gs* is the strength of the gap junctional coupling between myocyte and fibroblast.

The *MacCannell* fibroblast model equations^33^ were used to describe the time evolution of the fibroblast membrane potential $V_f$. The time evolution of the transmembrane potential for the *i*th fibroblast coupled to one myocyte is given by

3 $$\begin{aligned} C_f \times \frac{dV_{fi}}{dt} = -I_{ion_{fi}} + Gs (V - V_{fi}) \end{aligned}$$

with the ionic currents comprised of inward rectifying potassium current $I_{fK1}$, the time- and voltage -dependent potassium currents $I_{fKv}$, $I_{fNaK}$ a sodium-potassium pump current and a background sodium current $I_{bNa^+}$. For the myocytes we used the parameter set corresponding to *Shallow* restitution slopes (see Table 2, slope $=0.7$ for *Shallow* in ten Tusscher et al.^32^). We chose the *Shallow* restitution parameters for our study because for this slope the $TNNP-TP06$ tissue model does not initiate reentry. Thus any reentry observed in the study would be an outcome purely of the *M*–*F* coupling and not due to an inherent dynamical instability in the myocyte. The uncoupled fibroblast resting membrane potentials $V_{FR}$ were set to either $-24.5$ mV or $-49.0$ mV^12^. Most of the results described in the paper were obtained with $V_{FR}$ set to $-24.5$ mV. In order to test the effect of fibroblast resting potentials on our findings, a subset of the simulations were performed with $V_{FR} = -49.0$ mV. The different resting membrane potentials were obtained by shifting the gating variable voltage dependence of the time dependent potassium current^19^.

### Tissue model

The 2*D* simulations were performed using the monodomain formulation with the tissue discretised on a square lattice of size $N \times N$ (where $N = 200$ or $N = 400$).

4 $$\begin{aligned} \frac{\partial V}{\partial t} = \frac{-I_{ion}}{C_m} + \frac{\sum _{k=1}^{np} Gs (V_{fk} - V)}{C_m} + \nabla \cdot (D_{ij} \nabla V) \end{aligned}$$

The corresponding equation for the *k*th fibroblast unit coupled to *nm* grid points on the lattice is given by

5 $$\begin{aligned} \frac{dV_{fk}}{dt} = \frac{-I_{ion_{fk}}}{C_f} + \frac{\sum _{k=1}^{nm} Gs (V - V_{fk})}{C_f} \end{aligned}$$

The differential equations Eqs. (4) and (5) were solved using the forward Euler scheme and a standard five-point stencil was used for solving the Laplacian in Eq. (4). The space- and time- step were set to 0.25 mm and 0.01 ms respectively. $C_m$ and $C_f$ were the cell capacitance per unit surface area of myocyte and fibroblast set to 150 pF and 50 pF (corresponding to the larger myofibroblast^34^) respectively.

**Fig. 1:**
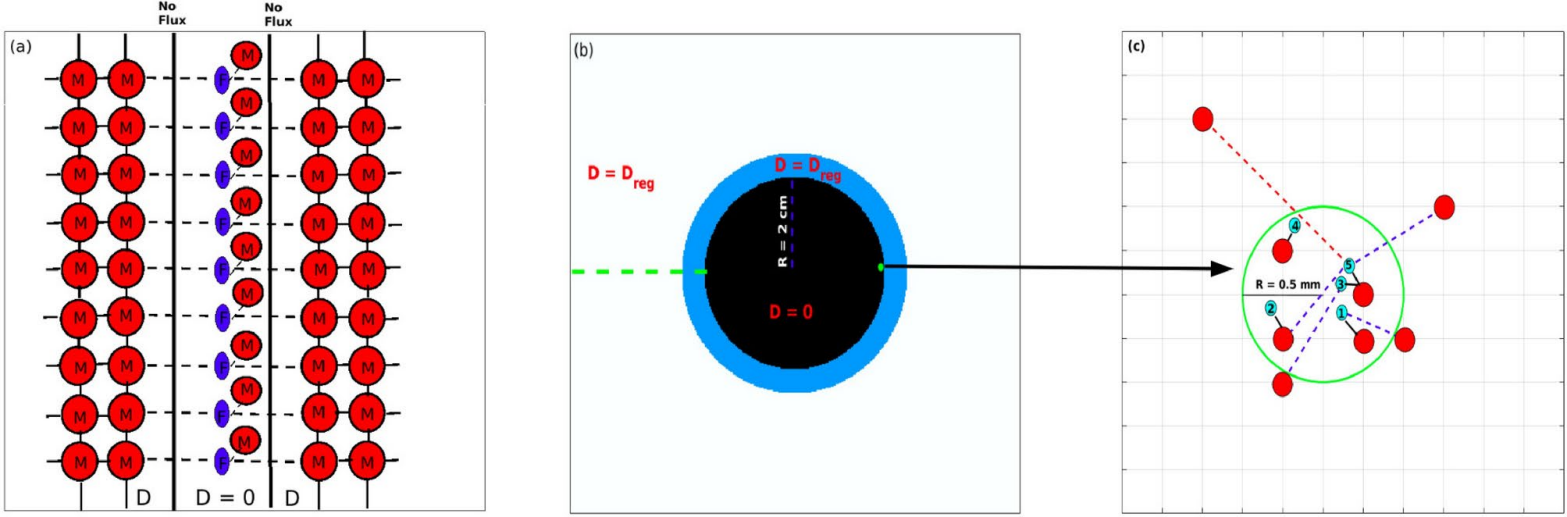
Schematic describing *M*–*F* links in 2*D* tissue. (**a**) *M*–*F* links (broken lines) coupling across a scar in a 2*D* tissue. (**b**) A circular scar region (black) of radius $R = 2$ cm with no coupling between cells $(D = 0)$ surrounded by a region of regular tissue (white) and border zone (blue) with $D = D_{reg}= 0.001~\textrm{cm}^2/\textrm{ms}.$ (**c**) Schematic showing the enlarged section of the scar region in green in (**b**) to describe both local and non-local *M*–*F* links in a domain of radius $R = 0.5$ mm. The red circles correspond to those myocytes units on the lattice grid that are coupled to fibroblast units. The blue circles indicate the 5 fibroblasts within the green circle that are attached to the myocyte units via both local and non-local *M*–*F* links. The local *M*–*F* links obeying constraint $L_{max}=0$ mm are drawn as solid black lines. The broken blue line (in combination with the local links) correspond to the *M*–*F* connections obeying constraint $L_{max} = 1.25$ mm. The broken red line (in combination with local links and broken blue links) correspond to the *M*–*F* connections obeying $L_{max} = 2.5$ mm.

In order to verify that *M*–*F* links can support propagation of conduction in tissue we first modelled a non-conducting scar region that diffusively separates tissue on its either side (Fig. 1a). The *M*–*F* links (broken lines in Fig. 1a) from the scar region also coupled tissue on either side of the scar acting as a conduction pathway.

We next modelled a circular scar tissue that diffusively uncoupled regions from within and outside the scar. Surrounding the scar tissue is a border zone that is connected to the tissue in the scar region purely via *M*–*F* links (see Fig. 1b). The layer of fibroblast units can be imagined to be on top of the scar tissue, providing electrical connections between the border zone and the scar. The scar region was constructed as a circle of radius $R = 2$ cm, consisting of active cells diffusively uncoupled from their neighbours. A subset of simulations were also performed for the case with inactive tissue in the scar (modelled by setting $I_{ion} = 0$ in the simulations). No-flux boundary conditions were implemented on the edges of the system domain as well as on the boundary of the scar region. The value of diffusion constant was set to $D_{ij} = 0$ in the scar region and $D_{ij} = 0.001~\textrm{cm}^{2}/\textrm{ms}$ everywhere else. A small section of the scar region adjacent to the boundary (green circle in Fig. 1b) is enlarged in Fig. 1c to illustrate the different types of links between the myocytes and fibroblast units. The black lines correspond to the local *M*–*F* links while the broken blue and red lines correspond to the non-local *M*–*F* connections.

Note that the border zone as constructed in this study is different from the way it has been represented in previous simulation studies. Conduction differences in the border zone are usually modelled using reduced tissue conductivity^35,36^ and the intrinsic electrophysiology of myocytes within the border zone modified by varying ionic currents^37–39^. However in the present study we have modelled the border zone keeping its diffusion environment and intrinsic electrical activity the same as that of rest of the tissue outside the scar. Here the change in the conduction properties of the border zone is modelled as an outcome of the *M*–*F* links that couple tissue inside the scar and the border zone. This model simplification allows us to investigate the effect of non-local coupling alone on the 2*D* tissue dynamics during rapid pacing. Using this setup we have verified our hypothesis that such long-range *M*–*F* links can promote reentry during rapid pacing. We generated rapid pacing waves at $T = 300$ ms by stimulating from one edge of the square domain. We have determined the effect of the long-range coupling on the local restitution properties and identified the parameters that initiate reentry. Our results are not critically dependent on the electrical activity of the cells in the scar or the fibroblast resting membrane potential, but are sensitive to the local distribution of *M*–*F* links in the border zone. We have also verified that the results obtained do not vary significantly for waves generated from point pacing.

### Simulating fibroblast mediated coupling in tissue

In order to simulate *M*–*F* coupling, we considered a layer of *np* fibroblast units directly attached to the scar region of the 2*D* myocardial lattice. Each lattice point on the grid represents a myocyte unit (a 0.25 mm square region containing around $3 \times 10$ myocytes). Each fibroblast unit (consisting of *Nf* fibroblasts connected in parallel^33,40^) is electrically coupled to one or more grid points on the lattice with a coupling strength *Gs*. We used $Nf = 4, 6$ and 8 in our simulations and determined that for the model parameters used, $Nf = 8$ was required to ensure conduction via *M*–*F* links. For all the results reported here we have used $Nf = 8$. For the results described here we have used $0 \le Gs \le 4$ nS; a range considered to be representative of the effect of fibroblasts in cell-cultures^19^.

While all fibroblast units were coupled to myocyte units in the scar, every grid point in the scar could have zero, one or more fibroblast units coupled to it. The fibroblast units themselves were not coupled to each other. Each fibroblast unit was coupled directly to one grid point with a strength $Gs = G_{loc}$; we refer to this myocyte unit as the proximal myocyte. The fibroblast unit may randomly also be connected to one or more distal grid points (myocyte units) up to an Euclidean distance of $L_{max}$ mm from the proximal myocyte unit with a strength $Gs = G_{Long}$. These myocytes are referred to as distal myocytes. While in general it is expected that $G_{long} \le G_{loc}$, in this paper we have only described the results for the case of $Gs = G_{loc} = G_{long}$. In other words, for the results considered here there is no spatial variation of gap-junctional coupling strengths. The number of grid points a given fibroblast unit can be connected to is drawn from a Poisson distribution with a parameter $\lambda$. The specific grid point to which a given fibroblast unit is coupled is chosen randomly with the constraint that it cannot be greater than $L_{max}$ mm away from the proximal myocyte unit on the lattice grid. For each set of parameters we simulated 5 realisations of the random distribution of *M*–*F* links (keeping $\lambda$, *np* and $L_{max}$ fixed). For the results reported here $L_{max}$ was set to 2.5 mm. It is important to note that the parameter $L_{max}$ represents the maximum Euclidean distance up to which a given fibroblast unit can couple to myocyte units on the grid. So for the distributions considered here, a given *M*–*F* link can only couple myocyte units that are located within a distance of 0–2.5 mm from each other. The sequence of steps to generate the *M*–*F* links is detailed in Algorithm 1.

**Algorithm 1:**
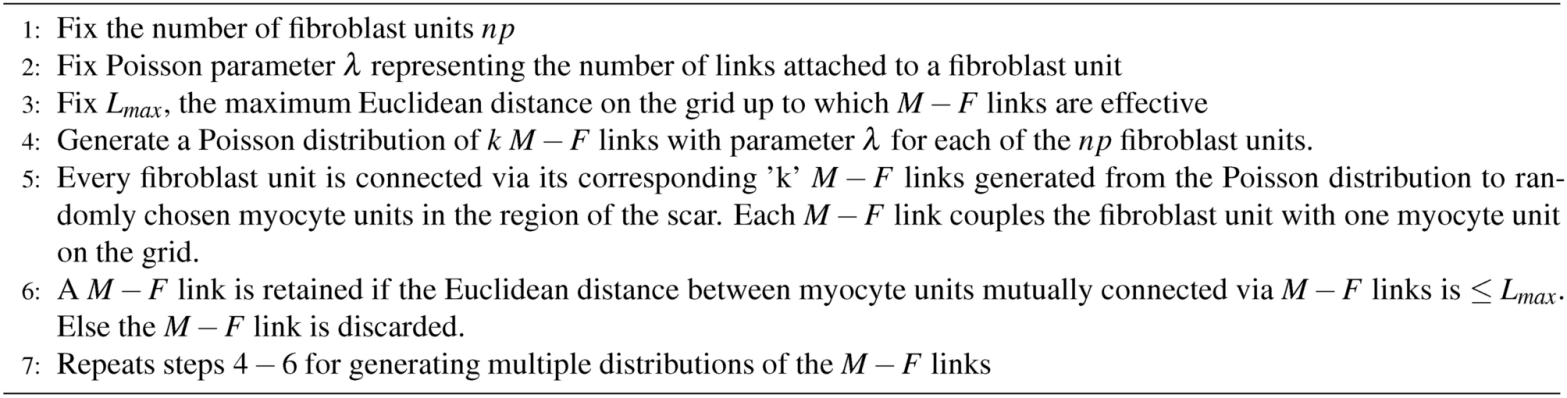
Algorithm for simulating long-range *M*–*F* coupling

**Fig. 2:**
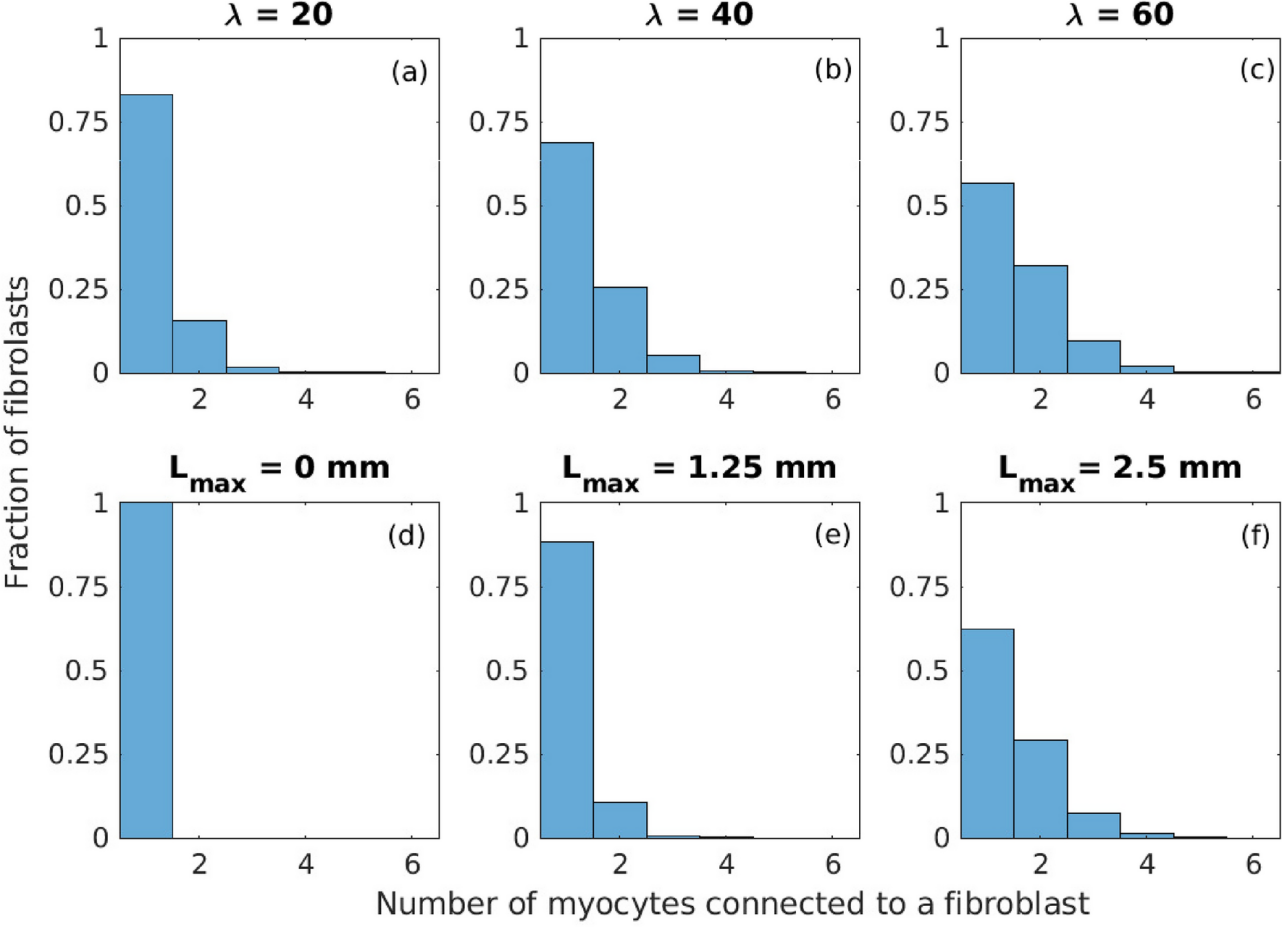
Effect of $\lambda$ and $L_{max}$ on the fraction of M–F links. The fraction of the fibroblast units gap-junctionally connected to a given number of myocyte units is plotted for different $\lambda$ values (**a**–**c**) and $L_{max}$ values (**d**–**f**). For the realisation of the *M*–*F* links shown here *np* was set to 20,000. For panels (**d**–**f**), $\lambda = 50$.

Figure 2 shows the distribution of the fraction of fibroblast units coupled to a given number of grid points for one spatial realisation of *M*–*F* links with the number of fibroblast units $np = 20{,}000$. Figure 2a–c shows the distribution for parameters $\lambda = 20$, 40 and 60 and $L_{max} = 2.5$ mm, while Fig. 2d–f shows the distribution for $L_{max} = 0$, 1.25 and 2.5 mm respectively for the case of $\lambda = 50$. We observed that irrespective of the parameter values, majority of the *M*–*F* links were connected to one fibroblast unit only. However with increase in both $\lambda$ and $L_{max}$, the fraction of fibroblast units coupled to more than 1 grid point increases. Similarly the maximum number of grid points coupled to any fibroblast unit increases with $\lambda$ and $L_{max}$ values. However for $L_{max} = 0$ mm, every fibroblast unit is coupled to one grid point only.

## Results

We first demonstrated conduction via *M*–*F* links in a 2*D* tissue of size $N \times N$ (with $N = 200$). Regions of active tissue on either side of a straight line scar (region with $D = 0$) are coupled via fibroblast units that are themselves attached to the myocytes in the scar(see Fig. 1a). The tissue on either side of the scar has normal diffusion properties. We observed that the conduction across the scar in the tissue scenario depended on the strength of the coupling. Weak coupling ($Gs = 2$ nS) for the *M*–*F* links as described in Fig. 1a do not result in conduction of the waves across the scar (see Supplementary movie SM1). Stronger coupling ($Gs = 4$ nS) resulted in a propagation of the wave across the scar with a delay in propagation across the scar boundary (see Supplementary movie SM2). This simple scenario of deterministic coupling links was used to illustrate the tissue conduction mediated purely via *M*–*F* links.

### Effect of long-range coupling on wave stability

**Fig. 3:**
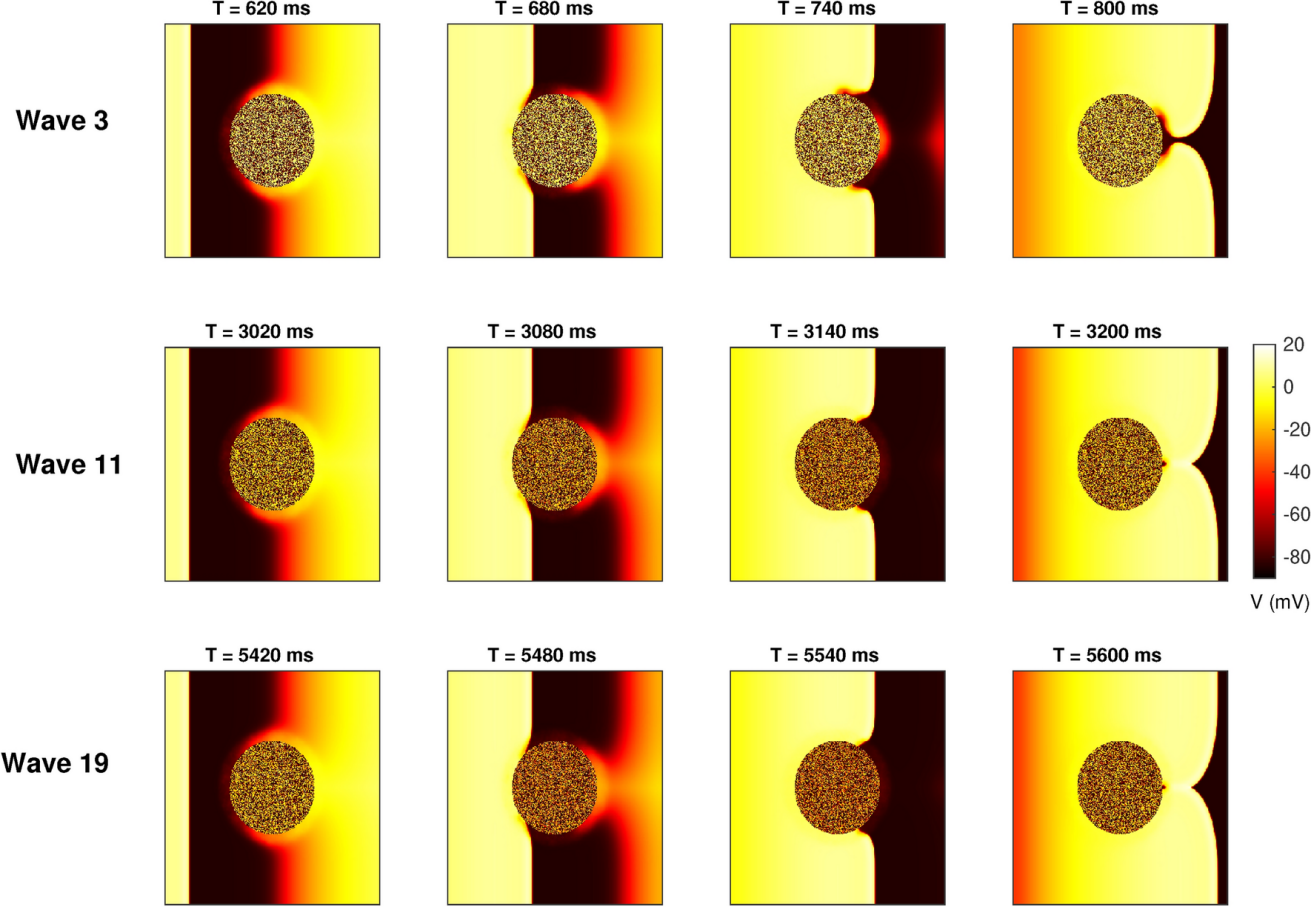
Pseudocolour image of the transmembrane potential *V* for the case of pacing at $T = 300$ ms and *M*–*F* coupling strength $Gs = 1.0$ nS resulting in no reentrant waves. The top, middle and bottom rows correspond to time snapshots for pacing waves 3, 11 and 19 respectively. The coupling parameters are $np=30{,}000$ and $\lambda = 50$.

We next investigated the effect of long-range *M*–*F* coupling on the stability of pacing waves. For this we generated rapid plane waves by stimulating one side of the 2*D* tissue at a period $T = 300$ ms for a duration of 6 seconds. We illustrate the effect of the long-range *M*–*F* coupling on the pacing waves by considering two coupling strengths $Gs = 1$ nS and $Gs = 4$ nS. Note that the *M*–*F* link distribution is the same for both the cases discussed below. Figure 3 shows the pseudocolour image of the transmembrane potential *V* for $Gs = 1$ nS over 6 seconds for one realisation of fibroblast distribution with $np = 30{,}000$ and $\lambda = 50$. For this coupling strength and distribution of *M*–*F* links, even 20 paced waves do not initiate any reentrant activity in the medium. While the velocity of the plane wave in the border zone is reduced, there is no significant change in the dynamics and the waves split and recombine behind the obstacle without initiating any retrograde dynamics (see Supplementary movie SM3).

**Fig. 4:**
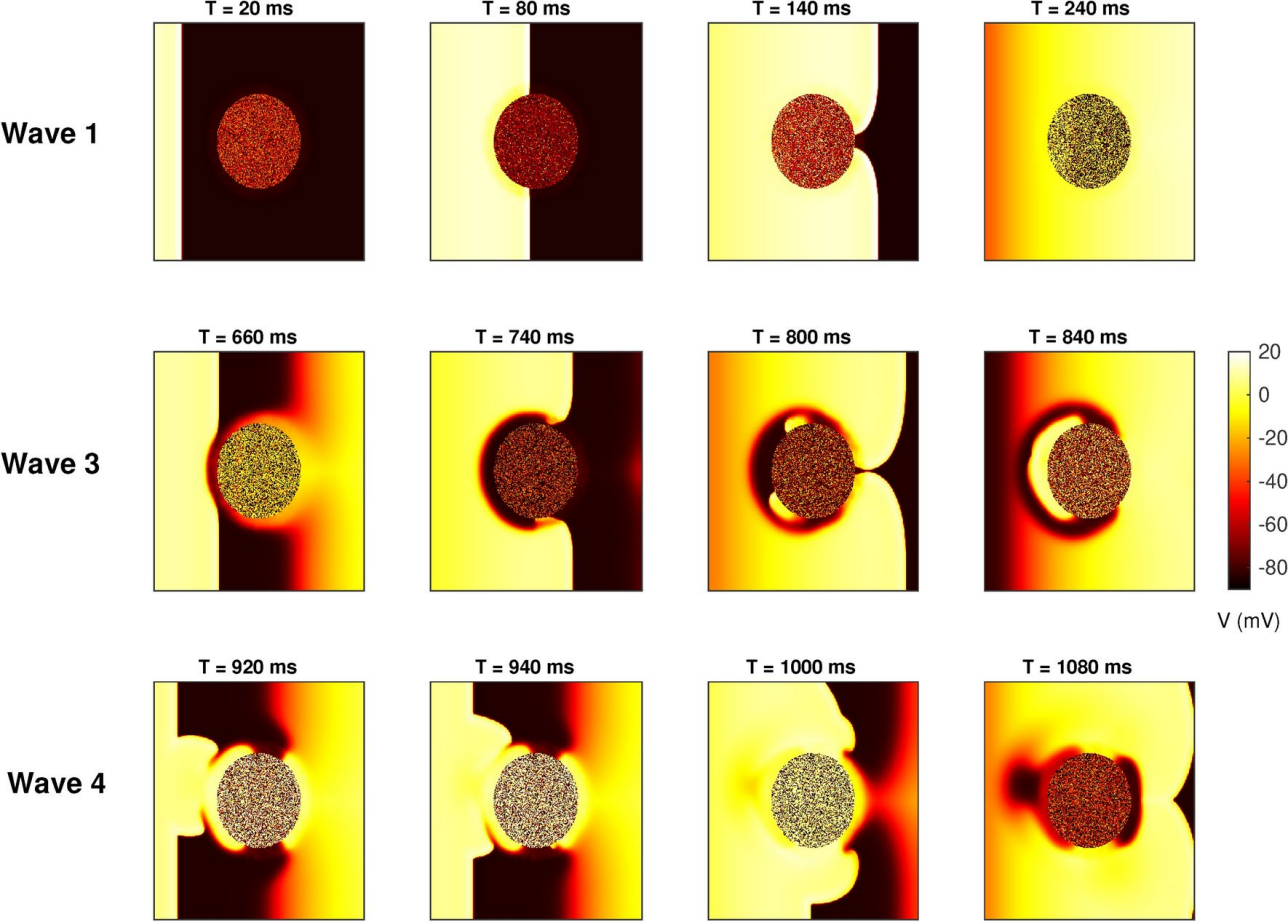
Pseudocolour image of the transmembrane potential *V* for the case of pacing at $T = 300$ ms and *M*–*F* coupling strength $Gs = 4.0$ nS that result in transient reentrant waves that invade the rest of the tissue. Top, middle and bottom rows correspond to time snapshots for pacing waves 1, 3 and 4 respectively. The coupling parameters are $np=30{,}000$ and $\lambda = 50$.

However increasing the coupling strength while keeping the same link distribution results in very different dynamics as seen in Fig. 4. It is observed that even as the third pacing wave approaches the scar, the border zone has not completely recovered and is locally inexcitable resulting in a zone of conduction block around the scar at $T = 660$ ms. This conduction block results in a significant slowing of the wavefront as it encircles the conduction block around the scar. Around $T= 740$ ms, as the wavefront propagates around the obstacle the border zone begins to recover. This results in a retrograde propagation of the wavefront as seen at $T = 800$ ms. This retrograde wave then collides with the next plane wave generated from the boundary ($T = 920$ ms) resulting in the formation of two curved wavefronts that then propagate into the rest of the tissue. The subsequent pacing waves results in further wave-breaks and reentrant waves (see Supplementary movie SM4).

In addition to the two dynamical scenarios described above, for some simulation parameters we have also observed short-lived transient reentrant waves that do not propagate beyond the border zone. Supplementary figure 1 shows an example of such a border zone reentry. While the *M*–*F* coupling produces a zone of conduction block ($T = 2220$ ms) as in the case of Fig. 4, the retrograde wave in the border zone at $T = 2260$ ms does not propagate outside it and is short-lived. Subsequent pacing produce similar short-lived waves that do not invade the rest of the tissue, for example waves 15 and 18 in Supplementary figure 1 (see Supplementary movie SM5).

**Fig. 5:**
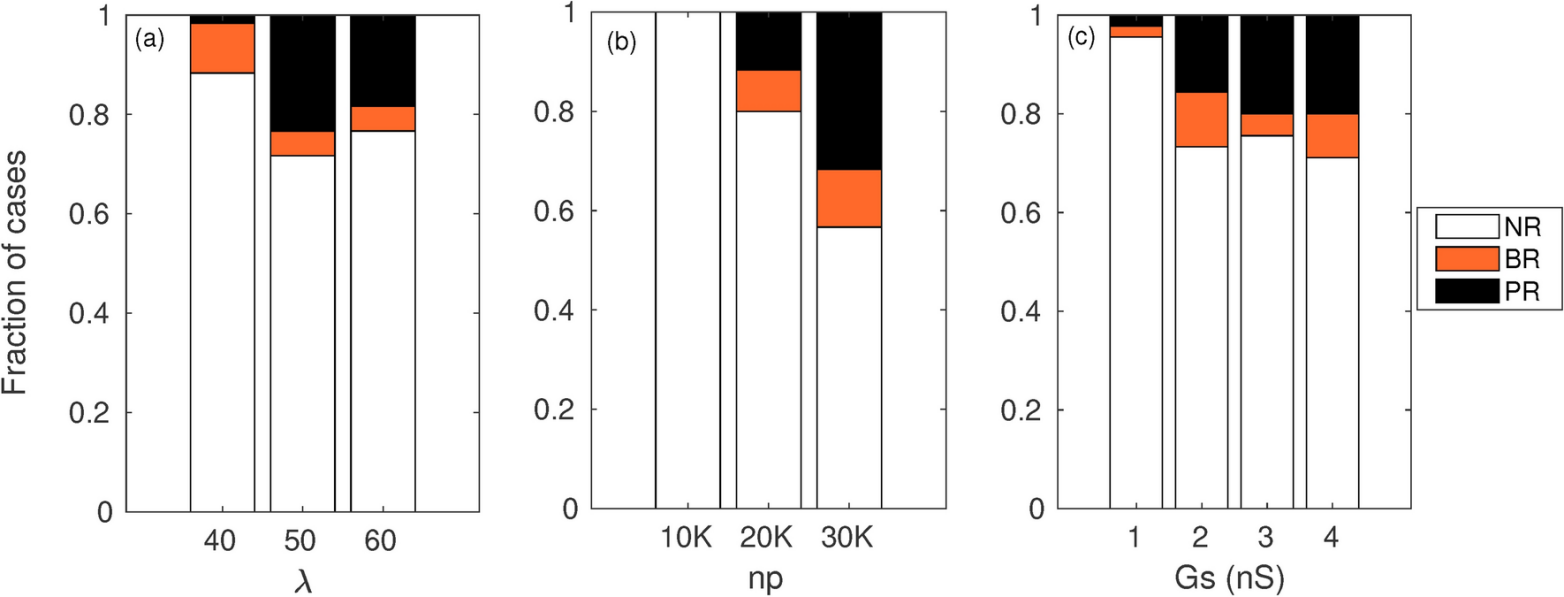
Effect of different parameters on the dynamics. Fraction of occurrence of each dynamical state *viz.,* no reentry (*NR*), reentry in the border zone (*BR*) and reentry propagating through the medium *PR* as a function of parameters *viz.,* $\lambda$ (**a**), *np* (**b**) and *Gs* (**c**) considered individually while summing over the other parameters.

More generally we performed simulations for all the different combinations of the parameters it viz., $\lambda$, *np* and *Gs*. For each combination of *np* and $\lambda$ values, simulations were performed for 5 spatial realisations of *M*−*F* links. Together with the 4 coupling strength values in all 180 simulations were performed and the dynamical regimes identified for each simulation. The distinct dynamical regimes identified include (i) no reentry (*NR*), (ii) short lived retrograde activity restricted to the border zone (*BR*) and (iii) reentrant waves that propagate through the medium and collide with subsequent pacing waves (*PR*). In Fig. 5, we have plotted the fraction of occurrence for each of the regimes in the 180 combinations of parameters. (Supplementary figure 5 shows individual histograms for each combination of parameters). While $\lambda = 40$ results in just 1 instance of *PR*, for $\lambda = 60$ nearly $20 \%$ of the simulations show *PR* (Fig. 5a). For $\lambda = 50$ more than $20 \%$ of the simulations resulted in *PR*. While for $\lambda = 40$ border zone reentry was observed in $10 \%$ of the simulations, the percentage of *BR* reduces to $5\%$ for higher $\lambda$.

While no reentry is seen for any parameter combinations with $np = 10{,}000$, larger values of *np* result in greater instances of both *BR* and *PR* (Fig. 5b). Most cases of reentry are observed for $np = 30{,}000$ with nearly $33 \%$ of the simulations showing *PR* and $11 \%$ showing *BR*.

For the case of coupling strength too an increase in occurrence of reentry is observed for an increase in *Gs* values (Fig. 5c). While for weak coupling ($Gs = 1$ nS) there is only 1 instance each of *BR* and *PR*, for both intermediate and stronger coupling strengths more than $15 \%$ of simulations show *PR* with the most instances observed for the strongest coupling. The occurrence of *BR* while more frequent for larger coupling strengths does not vary linearly with *Gs* values.

**Fig. 6:**
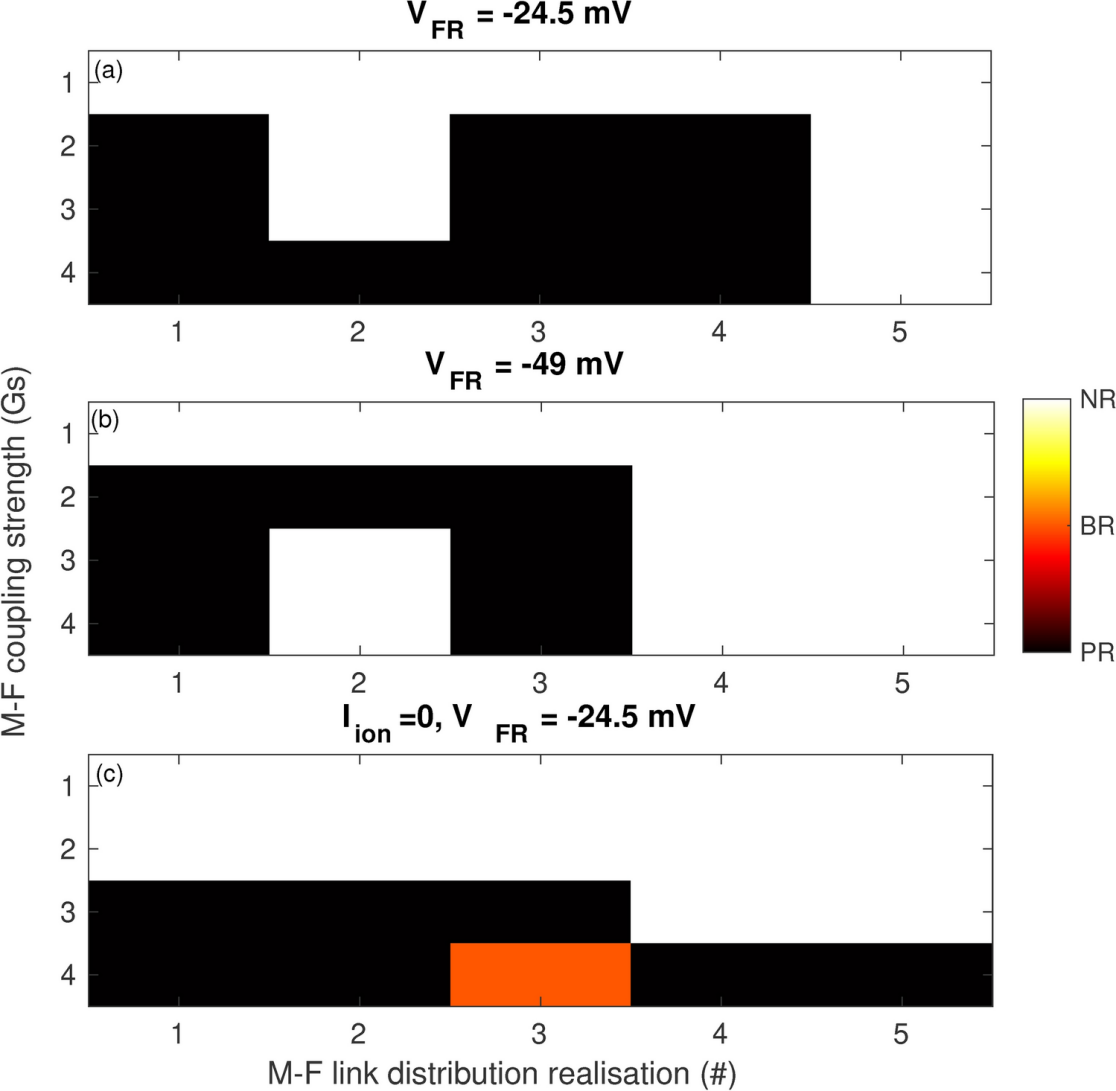
Dynamical regimes as a function of coupling strength (*Gs*). The conductance values resulting in the different dynamical regimes corresponding to no-reentry (*NR*), transient reentry in the border zone (*BR*) and complete reentry propagating through the tissue (*PR*) are identified for different spatial realisation of the *M*–*F* links. The fibroblast resting membrane potential is set to $V_{FR} = -24.5$ mV for panels (**a** and **c**) and $V_{FR} = -49.0$ mV for panel (**b**). The myocytes are active for the case shown in panels (**a**, **b**) and inactive for the case shown in panel (**c**). For all panels the parameters $np=30{,}000$ and $\lambda = 50$.

In Fig. 6a, we have highlighted the effect of the spatial distribution of *M*–*F* links using one combination of parameters($\lambda = 50$ and $np = 30{,}000$). The dynamical regimes are identified for different coupling strengths for all the 5 realisations of the *M*–*F* link distribution in the border zone. It is observed that for this parameter combination while generally higher coupling strengths do promote reentry, not all realisations of the *M*–*F* links result in a retrograde activity in the border zone. For example, irrespective of the coupling strength, realisation 5 does not promote reentrant activity even transiently. Thus the individual distribution of the connections in the border zone can determine the dynamics resulting from pacing.

In order to identify the effect of the fibroblast resting membrane potential on the dynamics, we ran a set of simulations for the parameters $\lambda = 50$ and $np = 30{,}000$ using the same 5 distributions of *M*–*F* links. Figure 6b describes the result for the effect of *Gs* on the dynamics for all the 5 realisations. It is observed that there are fewer occurrences of reentry for a more negative resting membrane potential (7 instances for $V_{FR} = -49$ mV compared to 10 instances for $V_{FR} = -24.5$ mV). Further the results of realisation 2 in Fig. 6b suggest that the onset of reentry is not linearly dependent on the strength of coupling *Gs*. While reentry is observed for $Gs= 2$ nS, both stronger and weaker coupling strengths do not promote reentry (see Supplementary movies SM6 ($Gs = 2$ nS) and SM7 ($Gs= 3$ nS)).

For all the results reported thus far the myocytes in the scar region were electrically active though uncoupled from each other. However it is physiologically likely that the tissue in the injured scar region becomes ionically inactive and does not generate an action potential. In order to verify the impact of the inactive tissue in the scar on the border zone dynamics we set $I_{ion} = 0$ for all the myocytes in the scar and performed simulations for the parameter set $\lambda = 50$ and $np = 30{,}000$. Figure 6c shows the dynamical regimes observed for the different realisations. While for this scenario we observed reentrant dynamics at larger values of *Gs*, unlike the case of active tissue in the scar (Fig. 6a) no reentry is observed for $Gs = 2$ nS. Furthermore unlike in Fig. 6a and b, *M*–*F* link realisation 5 promotes reentry (Fig. 6c).

### Mechanism of reentry

**Fig. 7:**
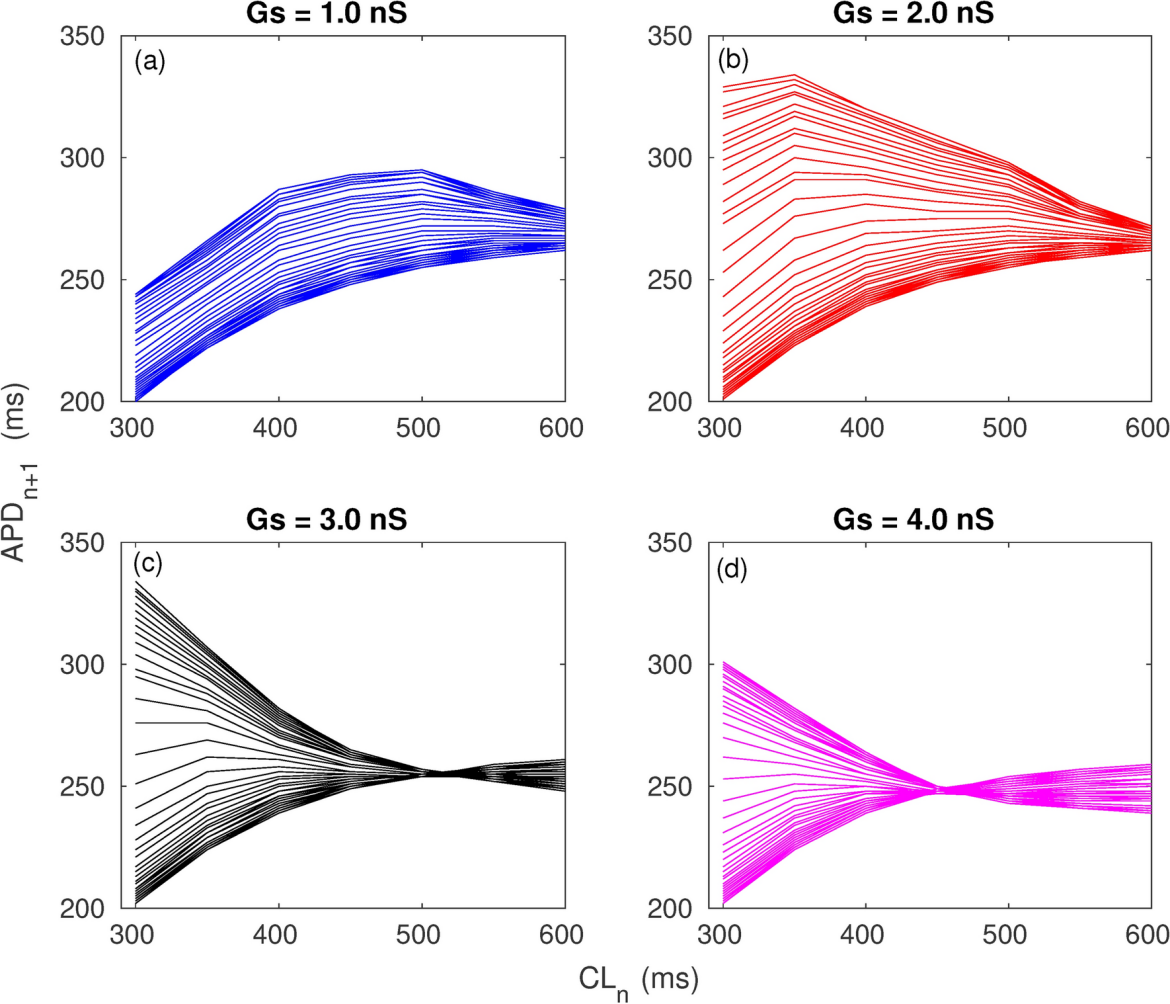
$CL_n$ vs $APD_{n+1}$. Pacing cycle length at the *n*th beat versus *APD* at the $(n+1)$th beat for points along the broken green line in Fig. 1b for different values of coupling strength. Other model parameters are $np=30{,}000$ and $\lambda = 50$.

**Fig. 8:**
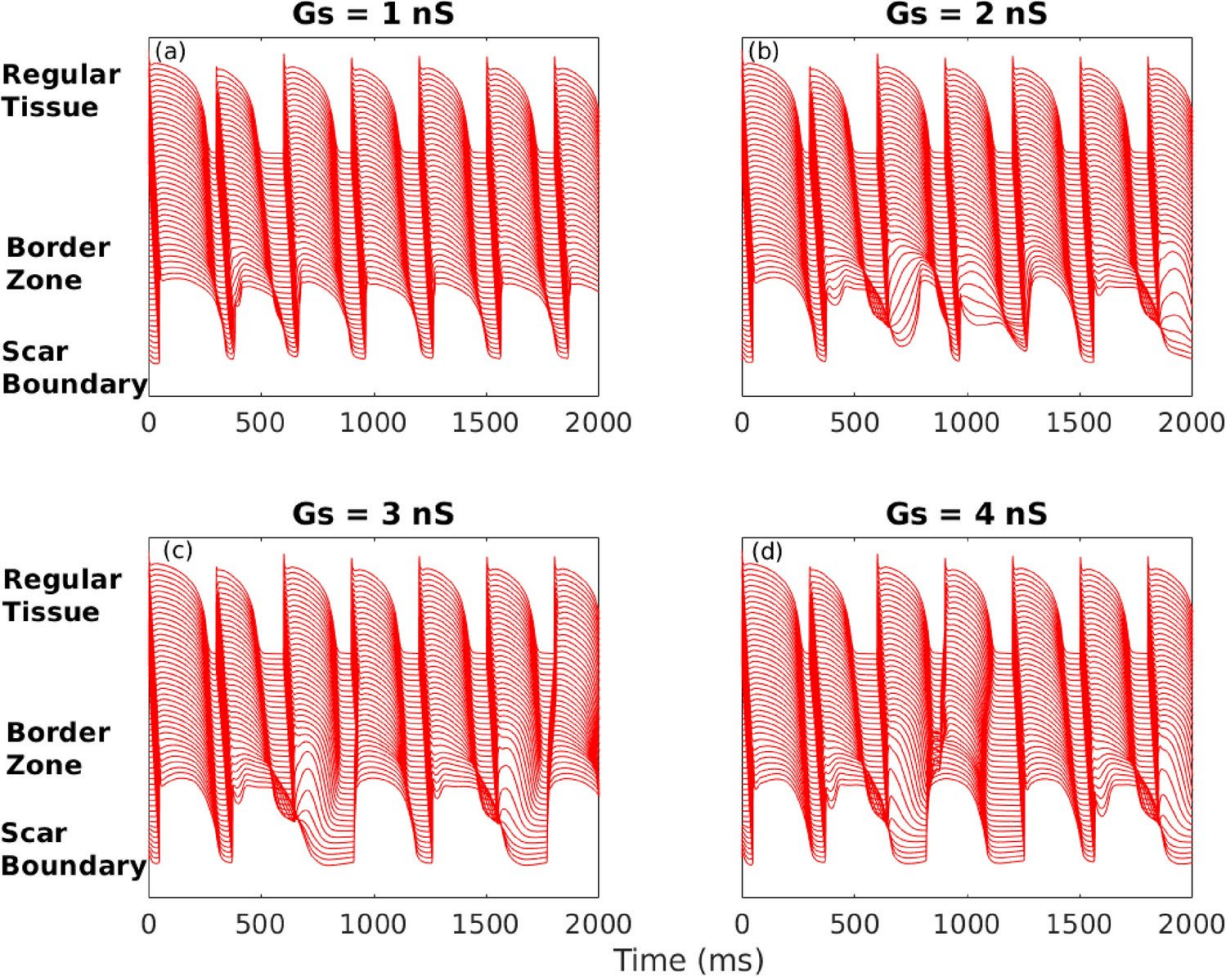
Space-time plots for different coupling strengths. The transmembrane voltage is plotted for cells along the broken line in Fig. 1b. For all panels $np=30{,}000$ and $\lambda = 50$.

In order to understand the mechanism underlying onset of reentrant waves we investigated the *S*1*S*2 restitution relation. For this we considered the parameters as in *M*–*F* Fig. 6a and link distribution corresponding to case 1. As observed in Fig. 6a while case 1 did not result in reentry for $Gs = 1$ nS for $Gs > 1$ it promoted reentry. We have plotted the action potential duration for the $(n+1)$th beat ($APD_{n+1}$) as a function of the pacing cycle length at the *n*th beat ($CL_n$) for the different coupling strengths in Fig. 7. The different lines correspond to local restitution curves for the different cells along the broken green line in Fig. 1b. It is observed that while the local restitution curves for the cells in the normal zone almost fall on top of each other, there is greater dispersion of the restitution curves for the cells in the border zone. The dispersion of $APD_{n+1}$ at the smallest $CL_n$ (which is $CL = 300$ ms, the period at which we are pacing the cell) is least ($\approx 45$ ms) for $Gs = 1$ nS and is maximum ($\approx 140$ ms) for $Gs = 2$ and $Gs = 3$ nS. For the case of the maximum coupling strength of $Gs = 4$ nS, the dispersion of $APD_{n+1}$ at the smallest $CL_n$ is $\approx 100$ ms. The spatial variation of *APD* in the border zone can be visualised via the space-time plots of the transmembrane potential shown in Fig. 8. The transmembrane potential is plotted for cells lying on the broken green line in Fig. 1b for the different coupling strengths. Maximal spatiotemporal variation of *APD* is seen in the border zone at coupling strengths $Gs = 2$ nS and $Gs = 3$ nS. For $Gs = 3$ nS, the large increase in *APD* following the 2nd beat in the cells close to the scar boundary results in conduction block of the 3rd wave. Another conduction block happens at the 6th wave following which we see retrograde propagation indicating reentry. A similar situation is observed for the case of $Gs = 4$ nS with a conduction block of the 3*rd* wave followed by a retrograde wave propagation indicating reentry. The mechanism described does not depend on the type of myocytes in the scar. Supplementary figure 4 compares the space-time plot for the case 1 with active (a–b) and inactive (c–d) myocytes. While conduction block is observed in panels (a, b, d) no such block is observed in panel (c) corresponding to $Gs = 2$ nS. See supplementary section for other examples (Supplementary figures 2 and 3) of the restitution curve and space time plots corresponding to cases 3 and 5 in Fig. 6a.

## Discussion

In this paper we have proposed a model to describe the effect of spatially non-local *M*–*F* coupling in diseased or injured hearts. Conduction between mutually uncoupled myocytes via fibroblasts are believed to occur across scars or ablation lines resulting in novel conduction pathways^27–29,41^. In our earlier study^30^, we had used simple 2-cell motifs to show that such fibroblast mediated coupling between mutually uncoupled myocytes can produce a range of dynamics including modification of action potential profiles and *APD*, delayed initiation of action potentials, synchronization of repolarization and excitation of resting cells. In this study we have extended the idea of fibroblast mediated coupling to tissue scale and developed a model to capture long-range effect of *M*–*F* links. We have hypothesised and verified *in-silico* that such non-local coupling of unconnected regions via fibroblasts can create a substrate to promote reentrant activity in injured fibrotic tissue during rapid pacing. The model allows to change the strength and number of non-local links which in turn determined the nature of the wave dynamics during pacing.

For the myocyte model we have chosen parameters corresponding to shallow restitution slope^32^ and to simulate the fibroblast dynamics we have used the *MacCannell* “active” model^33^ with modifications made to obtain different resting membrane potentials^19^. As we have considered a scenario representative of an injured tissue we set the fibroblast capacitance to $Cf = 50$ pF to capture effects corresponding to myofibroblasts.

We first demonstrated that conduction in tissue is possible via purely fibroblast mediated coupling for the geometry shown in Fig. 1a. We observed that depending on the strength of the *M*–*F* coupling the wave of excitation can be blocked or transmitted across the scar. Further it is observed that *M*–*F* coupling across the scar results in a delay in propagation (see Supplementary movies SM1 and SM2 respectively).

In order to simulate long-range coupling we coupled myocytes in the scar region with tissue outside the scar by *M*–*F* links (Fig. 1b,c). The *M*–*F* links were distributed randomly with a constraint on the distance up to which coupling could happen controlled by parameter $L_{max}$. The parameter $L_{max}$ determines the spatial extent of the border zone. $L_{max} = 0$ would mean there is no border zone and each fibroblast unit is coupled to only one grid point in the scar. While the $L_{max}$ parameter is physiologically determined by the size of the fibroblast and the length of its extensions in vivo, its actual value is experimentally unknown^2^. Freshly isolated fibroblasts are observed to be spherical in shape with typical diameters of about 7–9 $\upmu \textrm{m}$ and tend to have very few cytoplasmic processes^2^. However in vivo these fibroblasts form sheet like extensions and are observed to have several elongated cytoplasmic processes. While exact cell-size distribution of fibroblasts in vivo are not available, estimates of a total surface area of 1500 $\upmu \textrm{m}^2$ have been reported^24,25,41^. In the present study we have reported results for $L_{max} = 2.5$ mm, a value chosen to capture the variety of dynamics arising due to the effect of long-range coupling.

An important point to note is that while the conduction in the border zone is usually modelled by varying diffusion, the spatial variation of conduction in our study is determined by the recovery properties of the border zone. Thus the border zone is created dynamically and its spatial extent depends on the parameters *Gs*, $\lambda$ and the distance $L_{max}$ up to which the *M*–*F* links can exist. The cells in the border zone display a diverse profile of action potentials depending on the coupling strength, link density etc (Fig. 8).

In order to study the effect of the border zone on the stability of electrical waves we generated rapid plane waves of excitation by stimulating from the tissue boundary. The interaction of these waves with the border zone resulted in a range of dynamics including local conduction block followed by initiation of reentrant waves that either propagated through the rest of the tissue (Fig. 4) or were transiently active in the border zone (Supplementary figure 1).

To understand the mechanism underlying the onset of reentry due to fibroblast mediated long-range coupling, we have plotted the local restitution curves (Fig. 7) and the corresponding space-time picture (Fig. 8) for cells along the broken green line in Fig. 1b. As can be observed in Fig. 7, for the cases corresponding to $Gs = 2, 3, 4$ nS the myocytes in the border zone had a very large spatial variation of *APD* for pacing cycle length $CL_n = 300$ ms. The large spatial variation of *APD* in the border zone resulted in a continuous region of conduction block surrounding the scar as seen in Fig. 4 and Supplementary figure 1 (also see Supplementary movie SM4). The border zone acts as a substrate for the creation of conduction block and depending on the local distribution of *M*–*F* links a retrograde reentrant wave is produced. In comparison the same distribution of links produced a much smaller variation of *APD* for the case of $Gs =1$ nS (Fig. 3) and no conduction block and therefore no reentry was observed in the border zone (see Supplementary movie SM3). Similar dynamical behaviour are observed for other *M*–*F* realisations and parameters. For example in Supplementary figure 2 (panels a and b) describing the restitution curve for realisation 5 (Fig. 6a), the spatial variation of *APD* even for the strongest coupling is not large enough to create a region of conduction block that can act as a substrate for reentry. This can also be observed in the corresponding action potential profiles in the space-time picture (see Supplementary figure 3). Supplementary figure 2 (panels c and d) describes the restitution for *M*–*F* link realisation 3 (Fig. 6)a. It is observed that the spatial variation in *APD* at $CL_n = 300$ for $Gs = 4$ nS is sufficient to create a region of conduction block in the border zone. However this does not happen for the case of $Gs = 1$ nS where the dispersion of *APD* in the border zone at $CL_n = 300$ is much smaller. So for this realisation of *M*–*F* links $Gs = 4$ nS promoted reentry while $Gs = 1$ nS did not.

Figure 5 describes the effect of each of the parameters on the fraction of occurrence of the different dynamical regimes. We observed that the occurrence of reentry is a function of parameters such as *Gs*, $\lambda$ and *np* (in addition to the pacing cycle length *T* and scar size that have been fixed in this study in order to focus primarily on the long-range coupling and its effect on the initiation of reentry). An increase in the fraction of instances of reentry (both transient and propagating) is observed for an increase in the value of parameter *np*. For $np = 10{,}000$ no reentry was observed for any of the simulations. For the case of link density we observed that while $\lambda > 40$ resulted in more instance of reentry than $\lambda = 40$, $\lambda = 60$ showed a reduction in the cases of reentry compared to $\lambda = 50$. This suggests the existence of a lower and an upper bound on the *M*–*F* link density that can give rise to reentry. Fewer *M*–*F* links result in smaller modifications of *APD* in the border zone thus reducing the region of conduction block. A very large number of *M*–*F* links on the other hand can result in either a large current source or sink in the border zone again resulting in small changes in *APD* for the cells thereby preventing reentry. While there are very few instances of reentry for $Gs = 1$ nS, increased coupling strength increases the occurrence of both *BR* and *PR*. However there is no significant difference in the number of instances of reentry between $Gs =3$ nS and $Gs =4$ nS. Transient reentry (*BR*) that does not propagate outside the border tissue is observed for all values of coupling strength, with maximum instances observed for $Gs = 2$ nS. The transient border zone reentry is the result of local source sink mismatches arising due to the spatial variation in APD in the border zone for individual realisations and does not systematically vary with coupling strength.

Figure 6a describes the outcome of pacing for individual spatial distribution of *M*–*F* links for a particular combination of parameters. It can be observed that although reentry is generally more likely at larger *M*–*F* coupling strengths, the exact outcome is dependent sensitively on the spatial distribution of fibroblasts.

Our simulations indicate that the reentry mediated by the dynamic border zone does not depend upon the exact nature of the myocyte activity in the scar. Our model can describe a heart tissue in both early (scar with active myocytes) and late stages (scar with inactive myocytes) of injury. As can be observed in Fig. 6c, inactive myocytes in the scar can also result in conduction block and the mechanism leading to reentry (dispersion of *APD* in the border zone followed by initiation of retrograde activity due to local source-sink mismatches because of spatial variation of *M*–*F* links) is the same (see Supplementary figure 4 (panels c and d). Reducing the fibroblast $V_{FR}$ to a more negative value ($V_{FR} = - 49$ mV) changed the results of individual simulation. Reentry was still observed though in fewer instances (Fig. 6b).

While the effect of *M*–*F* coupling on wave dynamics in tissue has been studied extensively^22,23,42,43^, to the best of our knowledge this is the first study to discuss the long-range effects of fibroblast mediated coupling between diffusively uncoupled tissue. Long range coupling is more likely in diseased or injured hearts with a higher density of the larger myofibroblasts. Scenarios such as healing ablation scars^27^ or islands of myocytes trapped in scars^28,29^ can result in such long-range coupling.

We have identified the mechanism underlying the onset of conduction block and reentry in the tissue geometry. We have shown that conduction block is an outcome of the spatial variation in the *APD* border zone and onset of reentry depends critically on the local distribution of *M*–*F* links. Spatial heterogeneity in *APD* and recovery properties are known precursors to reentrant arrhythmia^37,44^. While the spatial variation in *APD* in the border zone results in a region of conduction block around the scar, the onset of reentry depends sensitively on the local variation of recovery which is a function of the local distribution of *M*–*F* links. Recent studies have highlighted the importance of fibrotic texture on the wave dynamics and stability^22,45–47^ The results of our study reiterate the importance of the local variation in spatial activity arising in this case out of local differences in the link density.

### Limitations and extensions

In our simulations we have not differentiated between the strength and nature of local and long range coupling. But in reality, coupling over longer distances will be weaker, *i.e.*, $G_{long}$ $\le$ $G_{loc}$. It has been experimentally observed that the *M*–*F* links are motile and location of myofibroblast contact changes with time^48^. The creation of a dynamic border zone and the motility of the *M*–*F* links is especially important during early stages of post-infarct healing. While the number of *M*–*F* links and their locations are fixed in our study, the model can be modified to capture the motility of the fibroblast contacts. The number of fibroblasts in a unit is an important factor that influences the myocyte action potential^33^. In the present study we have used a homogenised representation of *M*–*F* connections between myocycte and fibroblast units. This approach has enabled a tissue study that is computationally tractable, where the number of fibroblasts and connections can be systematically investigated to identify its effect on the border zone conduction properties. We have not incorporated mechanics of heart muscle contraction and the resultant changes to tissue geometry. Also mechanical-electrical feedback has been ignored. These are important considerations that will be incorporated in future studies. Furthermore in our study, we have only considered electrical coupling between myocytes and fibroblasts and have not included coupling between fibroblasts^29,49,50^ or ephaptic coupling^6^.

## Supplementary Information

Supplementary Information 1.

Supplementary Information 2.

Supplementary Information 3.

Supplementary Information 4.

Supplementary Information 5.

Supplementary Information 6.

Supplementary Information 7.

Supplementary Information 8.

## Data Availability

Our code is available at a Github public repository https://github.com/Sridhar2020/LongRangeCoupling.git.
