# Supplementary material for: The effect of non-local coupling of fibroblasts on pacing dynamics in a 2D tissue: a simulation study
Source: Sci Rep. 2025 May 8;15:16016. doi: 10.1038/s41598-025-99674-6 (PMC12062413; doi:10.1038/s41598-025-99674-6)
Supplement: Supplementary file 8 — Supplementary Information 8. [file 41598_2025_99674_MOESM8_ESM.pdf]

SUPPLEMENTARY FIGURES

The effect of non-local coupling of fibroblasts on pacing dynamics in a 2D tissue: a simulation study.

S. Sridhar and R. H. Clayton

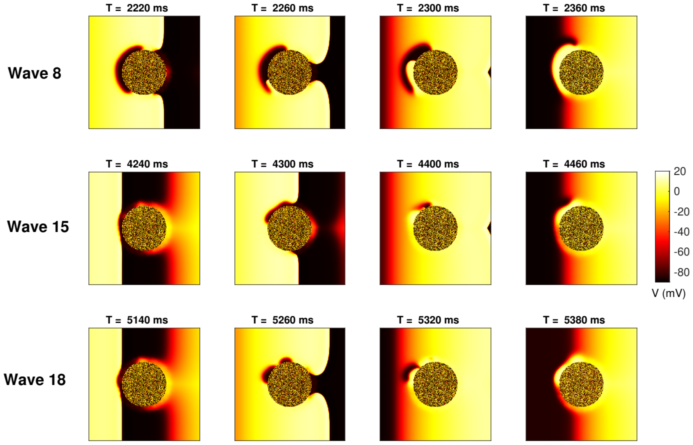

Supplementary figure 1: Pseudocolour image of the transmembrane potential V for the case of pacing at $T = 300$ ms and $M - F$ coupling strength $Gs = 2.0nS$ showing short-lived reentrant activity primarily in the border zone. Top, middle and bottom rows correspond to time snapshots for pacing waves $8, 15$ and 18 respectively. The M-F parameters for this case are $np = 20000$ and $\lambda = 50$.

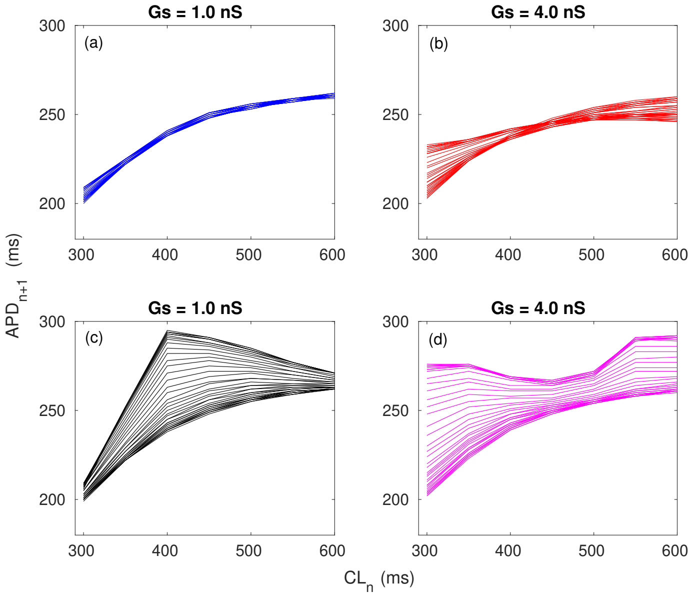

Supplementary figure 2: CL_n vs APD_{n+1}. Pacing cycle length (CL_n) vs APD_{n+1} (for points along the broken green line in main figure 1(b)) for two realisations of $M - F$ links in the border zone. Top row corresponds to the curve for one set of $M - F$ link distributions that does not result in reentry during pacing for both weak (a) and strong coupling strengths (b). Bottom row corresponds to a $M - F$ link distribution that does not initiate reentry at weak coupling (c), but promotes reentry at strong coupling (d). For all panels $np = 30000$ and $\lambda = 50$.

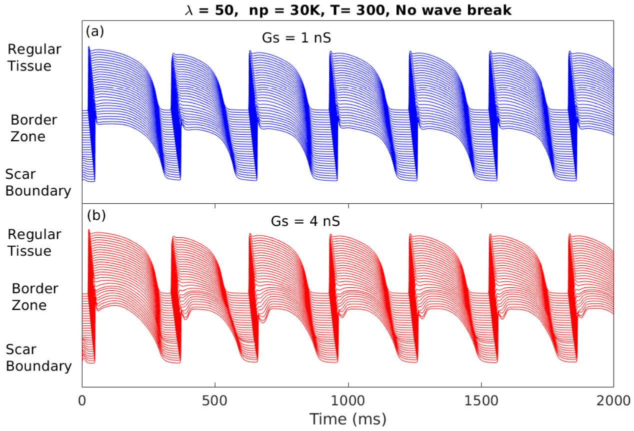

Supplementary figure 3: Space-time plots for a realisation that does not promote reentry. The transmembrane voltage is plotted for cells along the broken green line in main figure 1(b). For both panels $np = 30000$ and $\lambda = 50$.

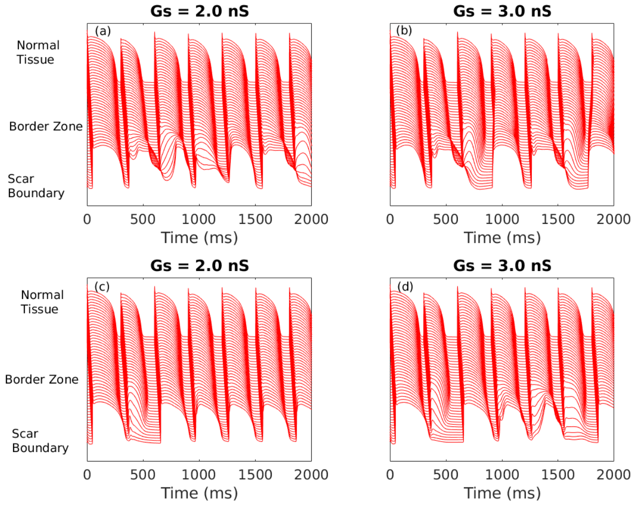

Supplementary figure 4: Comparing space-time plots for active and inactive myocytes in scar. Panels show the transmembrane voltage for cells along the broken green line in main figure 1(b). The myocytes in the scar are active (a-b) and inactive (c-d) respectively. For both panels $np = 30000$ and $\lambda = 50$.

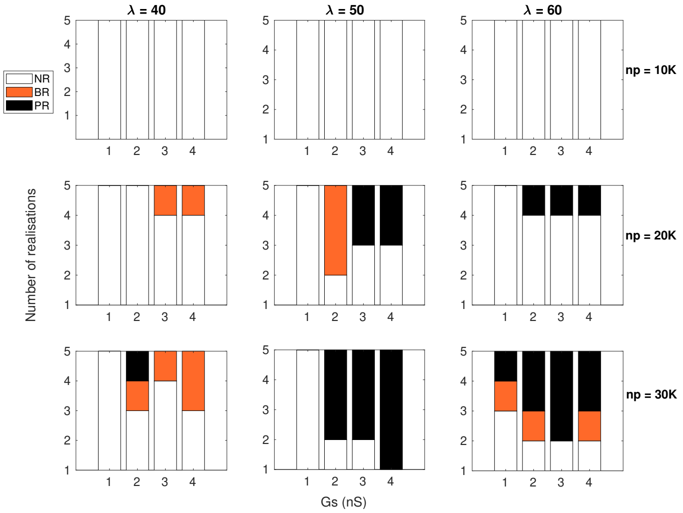

Supplementary figure 5: Histogram plots for the number of realisations describing the dynamical regimes NR (no reentry), BR (border zone reentry) and PR (propagating reentry) for different combinations of Gs, np and λ values.
